# Linearity extensions of the market model: a case of the top 10 cryptocurrency prices during the pre-COVID-19 and COVID-19 periods

**DOI:** 10.1186/s40854-021-00247-z

**Published:** 2021-05-18

**Authors:** Serdar Neslihanoglu

**Affiliations:** 1 Department of Economics and NIPE, University of Minho, Braga, Portugal; 2 Department of Statistics, Faculty of Science and Letters, Eskisehir Osmangazi University, Eskisehir, Turkey

**Keywords:** CAPM, COVID-19, Crypto Currency Index 30, Generalized additive model, Kalman filter, C10, C22, G01, G12

## Abstract

This research investigates the appropriateness of the linear specification of the market model for modeling and forecasting the cryptocurrency prices during the pre-COVID-19 and COVID-19 periods. Two extensions are offered to compare the performance of the linear specification of the market model (LMM), which allows for the measurement of the cryptocurrency price beta risk. The first is the generalized additive model, which permits flexibility in the rigid shape of the linearity of the LMM. The second is the time-varying linearity specification of the LMM (Tv-LMM), which is based on the state space model form via the Kalman filter, allowing for the measurement of the time-varying beta risk of the cryptocurrency price. The analysis is performed using daily data from both time periods on the top 10 cryptocurrencies by adjusted market capitalization, using the Crypto Currency Index 30 (CCI30) as a market proxy and 1-day and 7-day forward predictions. Such a comparison of cryptocurrency prices has yet to be undertaken in the literature. The empirical findings favor the Tv-LMM, which outperforms the others in terms of modeling and forecasting performance. This result suggests that the relationship between each cryptocurrency price and the CCI30 index should be locally instead of globally linear, especially during the COVID-19 period.

## Introduction

In recent decades, cryptocurrencies have witnessed spectacular development. They are growing rapidly and are used for many different applications in the economy due to their ability to facilitate electronic payments between individuals without the involvement of a (trusted) third party. Nakamoto (2008) was the first to document Bitcoin as the most well-known and prominent decentralized digital cryptocurrency based on blockchain technology. Bitcoin currently has the largest market capitalization among cryptocurrencies and has been widely used as a means of electronic payment in recent years due to the anonymity, safety, transparency, and cost effectiveness that it offers (Yermack 2013; Kim 2017; Yuneline 2019). The increasing popularity of Bitcoin has led researchers to develop other digital cryptocurrencies, such as Ethereum, XRP, and CRO. Presently, the digital crypto markets have grown rapidly in a short period of time; as of December 15, 2020, the global crypto market cap was $563.68 bn with 4028 cryptocurrencies (www.coinmarketcap.com). With the growing appeal of digital cryptocurrencies, finance analysts, economists, traders, and investors are focusing on predicting their future potential investment value. Therefore, numerous studies have recently been conducted to verify the influence of financial variables (e.g., gold prices, oil prices, currency exchange rates, commodities, stock market prices) on the fundamental and speculative value of cryptocurrencies, specifically with relation to their volatility as well as in terms of bubbles (e.g., Kondor et al. 2014; Kristoufek 2013; Ciaian et al. 2016; Bariviera et al. 2017; Zhu et al. 2017; Panagiotidis et al. 2018; Lahmiri et al. 2018; Nasir et al. 2019; Dennery 2020; Faghih Mohammadi Jalali and Heidari 2020; Hakim das Neves 2020; Huynh et al. 2020b, 2020c, 2020d; Makarov and Schoar 2020). In early 2017, the following group set out to objectively measure the overall growth and movement in the blockchain sector: Igor Rivin and Carlo Scevola (the team leaders of a group of mathematicians, fund managers, and quants); CS&P presidents and economists; as well as engineer Robert Davis. They designed the Crypto Currency Index 30 (CCI30) to track the top 30 cryptocurrencies by market capitalization, excluding stablecoins (www.cci30.com), and suggested that the CCI30 could serve as an investment tool for passive investors and investment managers. Limited literature on crypto markets has explored the use of the CCI30 index (Senarathne and Jianguo 2020; Pontoh and Rizkianto 2020; Petukhina, et al. 2020). To fill this gap, this research explores the stochastic behavior of the CCI30 index, especially for the purpose of helping crypto market policymakers and investors interested in portfolio diversification.

Over the last 9 months, the most prominent global health threat has been COVID-19, which was first detected in Wuhan, China on December 31, 2019, and was subsequently declared a global pandemic by the World Health Organization (WHO) on March 11, 2020 (WHO 2020). The pandemic spread rapidly throughout the world—despite quarantines, lockdowns, and social distancing—thereby upending the lives of millions of people. After COVID-19 was declared a global pandemic, the world economy was drastically affected. Worldwide, sales and production fell, companies became burdened financially, unemployment rose, and consumer behaviors changed (Lahmiri and Bekiros 2020a). The pandemic severely impacted financial markets, which, in turn, compelled many researchers to explore its effect on financial contagion and market stability (e.g., Akhtaruzzaman et al. 2020; Ali et al. 2020; Al-Awadhi et al. 2020; He et al. 2020; Okorie and Lin 2020; Sharif et al. 2020; Zaremba et al. 2020; Zhang et al. 2020; Shear et al. 2021). The rapid spread of COVID-19 in 2020 posed a serious threat to the crypto market as well. After the declaration of the pandemic, the largest 1-day fall in the price of Bitcoin (36%) occurred on March 13, 2020 (Yousaf and Ali 2021). Researchers started focusing on understanding the dynamics of the cryptocurrency market, especially the connections that have existed among the various cryptocurrencies during the COVID-19 crisis (e.g., Conlon et al. 2020; Conlon and McGee 2020; Corbet et al. 2020; Lahmiri and Bekiros 2020b; Mnif et al. 2020; Umar and Gubareva 2020; Yousaf and Ali 2020; James et al. 2021; Iqbal et al. 2021).

Several studies (e.g., Liu and Tsyvinski 2018; Rognone et al. 2020) have found that cryptocurrencies behave in a different manner from traditional assets, such as currencies, commodities, and equities. Furthermore, it was found that the returns in crypto markets are influenced by the enthusiasm of investors, which is, in turn, affected by unique and unusual events in the news. Considering the fact that the COVID-19 pandemic has been a very unique and unusual event given its unprecedentedness, researchers analyzed how the pandemic affected the cryptocurrency markets, especially in light of the disagreeing behavioral evidence. For example, Lahmiri and Bekiros (2020a) explored the evolution of informational efficiency in 45 cryptocurrency markets, including the CCI30 index and 16 international stock markets, from September 2019 to April 2020. They declared that cryptos showed more instability and more irregularity during the COVID-19 pandemic when compared to international stock markets. Mariana et al. (2021), on the one hand, evaluated the impact of COVID-19 on Bitcoin, Ethereum, gold, and the S&P500 from July 1, 2019, to April 6, 2020. All of the returns accrued during COVID-19 were found to be more volatile than the pre-COVID-19 period. They also declared that cryptos’ volatility is higher than that of both gold and the S&P500. Since valid, well-tested treatments and preventative strategies for COVID-19 are still lacking, these effects are expected to continue; thus, this research explores the impact of COVID-19 on the crypto market with the aim of providing investors and policymakers with a better understanding of the market dynamics of cryptocurrencies while allocating cryptos into their portfolios.

A widely used asset pricing model in portfolio applications that provides a guideline for crypto market investors is the capital asset pricing model (CAPM), which was independently developed by Sharpe (1964), Lintner (1965) and Mossin (1966) based on the Markowitz (1952) market portfolio model. The benchmark linear specification of the market model (LMM) is the data generating process (DGP) of the CAPM. In addition, the time invariant beta risk parameter, which is the slope coefficient of the CAPM, captures the global linearity between the financial asset returns and entire market returns and is commonly estimated via ordinary least squares (OLS). The linearity limitation of the CAPM beyond the benchmark LMM, however, has been explored by researchers due to rapidly changing global economic conditions (e.g., Kou et al 2014; Chao et al 2019) such as real-life credit and bankruptcy risks, trade-based money transaction methods, unemployment, inflation, and exchange rates. Jagannathan and Wang (1996) developed the time-varying linearity specification of the market model (Tv-LMM), which allows for a time-varying beta risk parameter known as the DGP of the conditional CAPM (C-CAPM). Here, Tv-LMM is designed in a state space model form via the Kalman filter algorithm (Kalman 1960) due to its performance (e.g., Mergner and Bulla 2008 in the pan-European industry; Zhang and Choudhry 2017 in European banks; Dębski et al. 2020 in the Polish, Czech, and Hungarian stock exchange; Neslihanoglu et al. 2020 in developed and emerging stock markets). While the Tv-LMM via the Kalman filter algorithm has been investigated extensively in several stock markets, firms, and industries, there is limited research on its use in crypto markets (e.g., Raimundo Júnior et al. 2020; Bianchi et al. 2020). Moreover, Neslihanoglu et al. (2017) proposed the generalized additive model (GAM) for flexibility in the rigid linearity shape of the LMM in developed and emerging stock markets, but no studies have used this approach to explore crypto markets. Given the lack of existing literature in this area, this research explores extensions of prior research on crypto markets.

The objective of the comparative analysis in this research is to shed light on the extensions of the LMM for modeling and forecasting cryptocurrency prices. Indeed, this is the first such comparison to be undertaken in the literature. To conduct the comparison, two extensions of the LMM model are investigated: the GAM, which allows for flexibility in the rigid linearity shape of the LMM, and Tv-LMM, in the mean reverting form of the state space model via the Kalman filter (KFMR) algorithm. This comparison is performed using daily data from two different time periods: pre-COVID19 (from January 1, 2019, to March 10, 2020) and during COVID-19 (from March 12, 2020, to November 1, 2020), specifically regarding the price index of 10 cryptocurrencies. The starting point of COVID-19 was chosen as March 11, 2020, following Mariana et al. (2021). This is based on the WHO declaring COVID-19 a global pandemic on that date (WHO 2020). The CCI30 served as a market proxy following Chowdhury et al. (2020), and the 1-month USD London Interbank Offered Rate (LIBOR) interest rate served as the risk-free rate proxy following Anyfantaki and Topaloglou (2018). For both time periods, 30 days forward are examined using 1-week and 7-day ahead predictions. The aforementioned models’ performance is compared using the mean absolute error (MAE), the mean square error (MSE), and a graphical summary.

This research contributes to the literature on cryptocurrencies in many ways with several first attempts. First, it investigates the impact of the COVID-19 pandemic on the financial stability of daily cryptocurrency prices based on modeling and forecasting using different time horizon forward predictions. Second, it evaluates the effectiveness of the LMM, which allows the time invariant beta risk to capture the global linearity between cryptocurrency returns and the CCI30′s returns—something that is commonly estimated via OLS. Third, it evaluates the nonlinearity extension of the market model via GAM underpinning the polynomial model on the cryptocurrency price. Next, it evaluates the local linearity extension of the market model in the state space model form via the Kalman filter algorithm on the cryptocurrency price while also accounting for the time-varying behavior of the beta risk parameter of the cryptocurrency price. Finally, it evaluates the impact of COVID-19 on the stochastic behavior of the time-varying beta risk of cryptos with the aim of providing investors with a quantifiable metric with which to build their crypto portfolios and to better understand the possible risks and rewards of each cryptocurrency.

The rest of this research is laid out in the following way. Second section outlines the overview of data, while third section provides the detailed methodologies of the proposed models. Fourth section presents the empirical outcomes from the comparison of the aforementioned models, while also showing the parameter estimation in the best model. Finally, fifth section summarizes the research.

## Data description

The daily data of this research span two time periods: pre-COVID-19 (from January 1, 2019, to March 10, 2020) and COVID-19 (from March 12, 2020, to November 1, 2020). The data pertain specifically to the price index of the 10 cryptocurrencies. The CCI30, which tracks the top 30 cryptocurrencies by adjusted market capitalization (www.cci30.com), serves as the market proxy in this research. The main criteria of selecting these cryptocurrencies were that they had the highest market capitalizations in all 30 cryptocurrencies on November 1, 2020, and that they were also continuous listings and on the CCI30 during the pre-COVID-19 and COVID-19 periods as defined by March 11, 2020 [the day the WHO declared COVID-19 as a global pandemic (WHO 2020)]. As suggested by Alexander and Dakos (2020) and Huynh et al. (2020a), the validity of the data set was checked using different data sources for crypto prices. Table 1 provides an overview of the variables, their abbreviations, and their data sources.

**Table 1:** Variables, abbreviations, and sources

Variables	Abbreviation	Source
*Cryptocurrencies Index 30*	CCI30	www.cci30.com
*Cryptocurrencies*		
Bitcoin Price Index	BPI	www.coinmarketcap.com
Ethereum Price Index	ETH	www.coinmarketcap.com
XRP Price Index	XRP	www.coinmarketcap.com
Bitcoin Cash Price Index	BCH	www.coinmarketcap.com
Binance Coin Price Index	BNB	www.coinmarketcap.com
Chainlink Price Index	LINK	www.coinmarketcap.com
Crypto.com Coin Price Index	CRO	www.coinmarketcap.com
Litecoin Price Index	LTC	www.coinmarketcap.com
Bitcoin SV Price Index	BSV	www.coinmarketcap.com
Cardano Price Index	ADA	www.coinmarketcap.com
*Risk-free rate*	*R*_*f*_	www.macrotrends.net

The daily data returns of the 10 cryptocurrencies and the CCI30 as the log difference of the daily closing price index in USD are determined as follows.

1 $${R}_{it}=\text{log}\left({P}_{it}\right)-\text{log}\left({P}_{i t-1}\right)$$

where *i* = 0*,*1*,…,*10 and *t* = 2*,…,T.* Here, *i* = 0 refers to the CCI30 (${R}_{mt}$) and ${R}_{it}$ with $1\le i\le 10$ referring to each cryptocurrency. *P*_*it*_ is the daily closing price index of those in day *t.* The 1-month USD LIBOR interest rate in percentage per annum serves as the risk-free rate (${R}_{ft}$) proxy over time *t*.

Table 2 displays descriptive statistics for the returns of CCI30, the 10 cryptocurrencies, and the 1-month USD LIBOR interest rate during the pre-COVID-19 and COVID-19 periods. Table 2 provides the key empirical features of the data. The mean returns of cryptocurrencies (0.00154 (average)) and the CCI30 (0.00076) during the COVID-19 period were lower than that of the cryptocurrencies (0.00412 (average)) and the CCI30 (0.00373) during the pre-COVID-19 period. This means that investors realized greater financial gains during the COVID-19 period than during the pre-COVID-19 period. Moreover, the standard deviations (unconditional volatility) of returns in the cryptocurrencies (0.05233 (average)) and in the CCI30 (0.03658) in the pre-COVID-19 period are greater than those of returns in the cryptocurrencies (0.04314 (average)) and in the CCI30 (0.03373) during the COVID-19 period. This suggests that cryptocurrencies and the CCI30 were deemed less risky investments during the COVID-19 period when compared to the pre-COVID-19 period, when the risk is measured by unconditional volatility. These results suggest that the cryptocurrencies have been quite affected by the COVID-19 global pandemic.

**Table 2:** Descriptive statistics of daily data during the pre-COVID-19 and COVID-19 periods

Period	Pre-COVID-19						
Variables	Mean	Std. Dev	Skewness	Kurtosis	JB	LB(21)	ADF
*CCI30*	0.00076	0.03658	− 0.2901	6.9718	291.351*	26.513	− 7.079*
*Cryptocurrencies*							
BPI	0.00166	0.03433	0.2017	7.2904	335.815*	23.294	− 7.163*
ETH	0.00082	0.04204	− 0.4421	6.5166	237.758*	25.820	− 6.963*
XRP	− 0.00125	0.03740	0.3718	7.9525	453.535*	28.735	− 7.462*
BCH	0.00115	0.05420	0.7212	11.2331	1263.398*	19.099	− 6.642*
BNB	0.00235	0.04318	− 0.0267	5.2105	88.413*	19.186	− 7.316*
LINK	0.00602	0.06279	1.5492	11.8958	1604.640*	16.164	− 6.392*
CRO	0.00191	0.07439	4.5874	50.2965	41,973.972*	62.871*	− 6.379*
LTC	0.00104	0.04810	0.5105	7.3985	368.709*	25.335	− 7.587*
BSV	0.00177	0.08051	4.1103	43.6248	31,066.326*	23.477	− 5.971*
ADA	− 0.00006	0.04633	− 0.093	4.8566	62.955*	26.381	− 7.076*
*Average*	*0.00154*	*0.05233*	*1.1490*	*15.6275*			
*R*_*f*_	0.00008	0.00001	− 0.7352	3.1033	39.289*	6895.346*	

Period	COVID-19						
Variables	Mean	Std. Dev	Skewnesss	Kurtosis	JB	LB(15)	ADF
*CCI30*	0.00373	0.03373	0.0872	7.0417	159.568*	33.923*	− 6.329*
*Cryptocurrencies*							
BPI	0.00434	0.03172	0.7581	7.7928	246.381*	29.645*	− 5.733*
ETH	0.00539	0.04317	0.3346	5.9994	92.082*	32.594*	− 6.652*
XRP	0.00231	0.03328	0.4664	6.2167	109.371*	25.214	− 5.200*
BCH	0.00241	0.04053	− 0.002	8.8802	337.128*	23.625	− 6.298*
BNB	0.00464	0.04265	− 0.0035	7.0201	157.569*	52.339*	− 6.195*
LINK	0.00736	0.06448	0.4565	5.4356	65.967*	19.756	− 5.188*
CRO	0.00440	0.03741	0.1833	5.0457	42.115*	24.672	− 4.902*
LTC	0.00251	0.04044	0.066	7.6653	212.376*	25.204	− 6.149*
BSV	0.00177	0.04563	1.0059	11.3505	719.338*	17.092	− 6.419*
ADA	0.00603	0.05204	0.3828	5.0043	44.881*	21.441	− 5.610*
*Average*	*0.00412*	*0.04314*	*0.3648*	*7.0411*			
*R*_*f*_	0.00001	0.00001	1.7173	4.255	130.373*	2772.950*	

Std. Dev. denotes the annualized standard deviations of the data. *JB* denotes the Jarque–Bera statistics for the null hypothesis of normally distributed cryptocurrency returns. *LB* denotes the Ljung–Box statistics for the null hypothesis of no autocorrelation in the cryptocurrency returns. *ADF* denotes the Augmented Dickey Fuller test statistics for the null hypothesis of non-stationary cryptocurrency excess returns. *denotes the appropriate null hypothesis being rejected at the 5% significance level

The distributions of cryptocurrencies exhibit positive average skewness (1.1490; 0.3648) in the pre-COVID-19 and COVID-19 periods, while the returns of CCI30 (− 0.2901; 0.0872) and *R*_*f*_ (risk-free rate) (− 0.7352; 1.7173) exhibit negative skewness in the pre-COVID-19 period but positive skewness in the COVID-19 period. This signifies that there were frequent small dips and a number of massive increases in returns in all variables during the COVID-19 period. In addition, the return distributions for all cryptocurrencies, CCI30, and *R*_*f*_ are leptokurtic, which suggests the larger tails when compared to a normal distribution and a higher probability for immense results for all variables. This implies that there was a higher possibility for extreme financial gains or losses in investment cryptocurrency returns during the pre-COVID-19 and COVID-19 periods. The normality of all variables is also rejected at a significance level of 5% via the Jarque–Bera test. The null hypothesis of no autocorrelation is rejected at the 5% significance level only for CRO during the pre-COVID-19 period and for CCI30, BPI, ETH, and BNB during the COVID-19 period. According to the ADF test, the time series of the 10 cryptocurrencies and the CCI30 market excess returns was stationary during the pre-COVID-19 and COVID-19 periods.

To sum up, the key characteristics of this research data are positive means, volatility, asymmetrical (left- and right-skew), and leptokurtosis (fat tails) for both time periods. These features match those regularly reported by cryptocurrency studies, especially Catania et al. (2019) and Yousaf and Ali (2020). These results justify the consideration of extending the linearity between each cryptocurrency with CCI30 for both time periods.

**Fig. 1:**
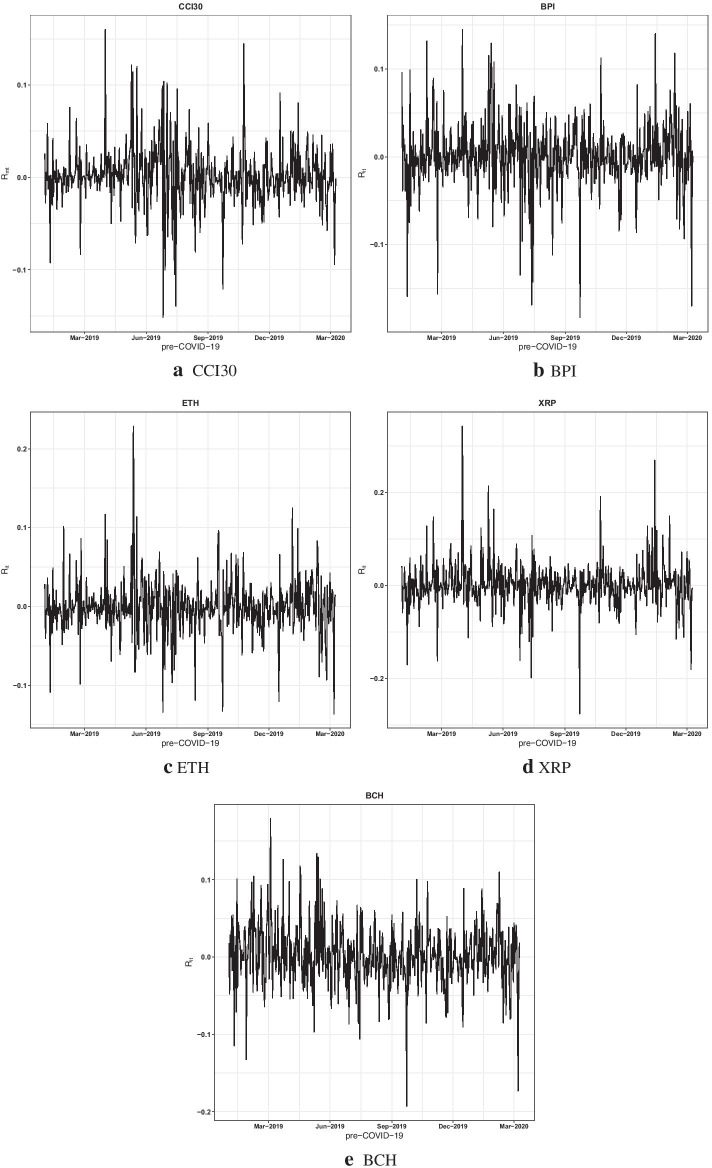
Time series plots of the CCI30 and four cryptocurrencies’ returns during the pre-COVID-19 period

**Fig. 2:**
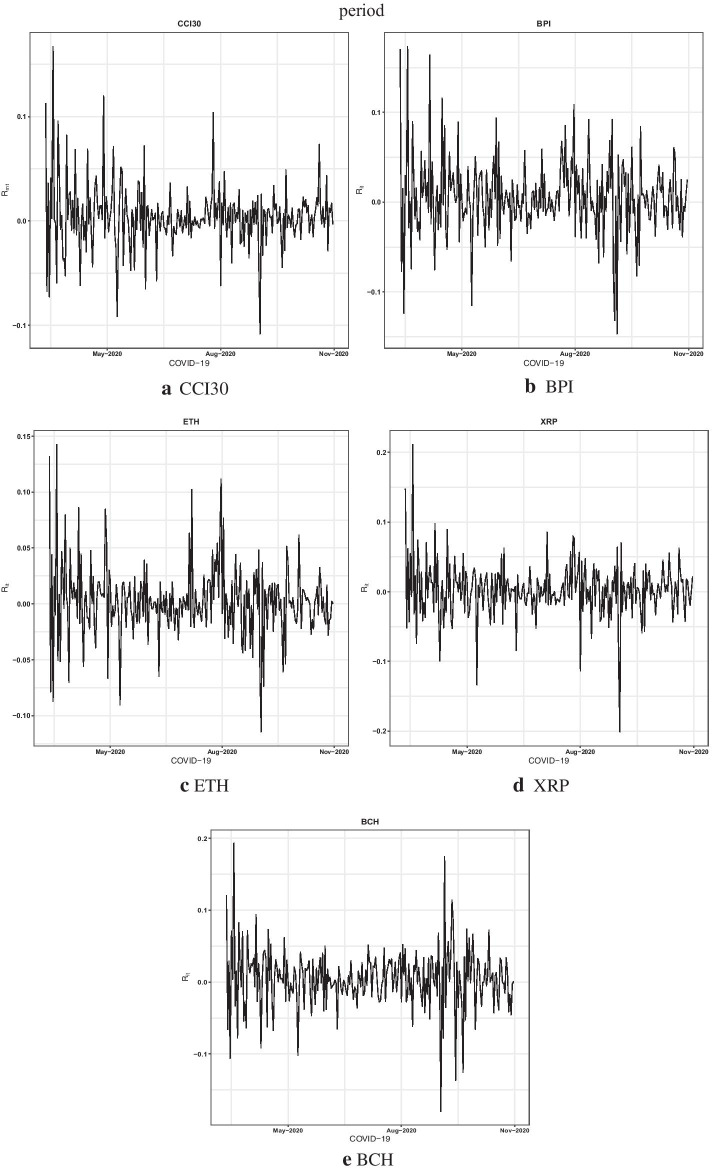
Time series plots of the CCI30 and four cryptocurrencies’ returns during the COVID-19 period

For the sake of brevity, the time series plot of the top four cryptocurrencies (BPI, ETH, XRP, and BCH) and the CCI30 returns during the pre-COVID-19 and COVID-19 periods are represented in Figs. 1 and 2, respectively. These figures provide some key insights. The large fluctuations in the returns of the CCI30 and cryptos are a common characteristic during both periods. After the declaration of COVID-19 as a global pandemic (on March 11, 2020), the largest 1-day fall in the price of Bitcoin was 36% on March 13, 2020 (Yousaf and Ali 2021); the following week showed an increase in fluctuations of returns in the cryptos, as can be observed in Fig. 2.

## Methodology

### Linear market model (LMM)

The benchmark LMM was independently developed by Sharpe (1964), Lintner (1965), and Mossin (1966) and is known as the DGP of the CAPM. This model is defined as follows.

2 $${R}_{it}-{R}_{ft}={\alpha }_{i}+{\beta }_{im}\left({R}_{mt}-{R}_{ft}\right)+{\varepsilon }_{it} \;\;\;\;\;\; {\varepsilon }_{it} \sim N\left(0,{\sigma }_{i}^{2}\right)$$

Let ${R}_{it}$ be the returns in cryptocurrency *i* $\left(i=1,\cdots ,10\right)$, ${R}_{mt}$ be the returns in the CCI30 index, and ${R}_{ft}$ be the risk-free rate at time *t *$\left(t=1,\cdots ,T\right)$*.* Additionally, residuals are ${\varepsilon }_{it}$ with ${\varepsilon }_{it} \sim N\left(0, {\sigma }_{i}^{2}\right)$ and $E\left({\varepsilon }_{it}{\varepsilon }_{kt}\right)=0$, for $i\ne k$ and $E\left({\varepsilon }_{it}{\varepsilon }_{i i+j}\right)=0$*,* for $j>0$. Here, ${\alpha }_{i}$ is the regression intercept, and ${\beta }_{im}$ is the regression slope and accounts for the time invariant beta risk. OLS, which is briefly outlined by Wood (2006), is used to estimate the coefficients of Eq. (2). Note that the analyses of OLS were computed using the *lm* function in the *R* software (R Core Team 2018).

### Generalized additive model (GAM)

Developed by Hastie and Tibshirani (1990), the GAM is used to evaluate whether the rigid parametric shapes of the LMM (Eq. 2) are too restrictive in this research. The GAM extension of the LMM is given as follows.

3 $${R}_{it}-{R}_{ft}={\alpha }_{i}+{f}_{i}\left({R}_{mt}-{R}_{ft}\right)+{\varepsilon }_{it} \;\;\;\;\;\; {\varepsilon }_{it} \sim N(0,{\sigma }_{i}^{2})$$

Here, ${\varepsilon }_{it} \sim N\left(0, {\sigma }_{i}^{2}\right)$ with $E\left({\varepsilon }_{it}{\varepsilon }_{kt}\right)=0$, for $i\ne k$ and $E\left({\varepsilon }_{it}{\varepsilon }_{i i+j}\right)=0$, for $j>0$. Let ${f}_{i}\left({R}_{mt}-{R}_{ft}\right)$ be a smooth function of ${R}_{mt}-{R}_{ft}$, whose shape is estimated from the data. It has a potentially non-linear relationship with ${R}_{it}-{R}_{ft}$. Here, the GAM provides a nonlinearity extension of the market model with ${\left({R}_{mt}{-R}_{ft}\right)}^{\tau }$, where $\tau$ takes any positive values instead of an integer that represents the polynomial extension of LMM (Eq. 2). The shape of the fitted model via GAM will be estimated from the data itself (Simpson 2018). The GAM parameter estimation procedure is briefly outlined by Wood (2006). Note that the analyses of GAM were computed using the mgcv package in the R software (R Core Team 2018).

### Time-varying linear market model (Tv-LMM)

The time-varying extension of LMM, which allows for the time-varying beta risk parameter and is also known as the DGP of C-CAPM developed by Jagannathan and Wang (1996), is referred to here as the Tv-LMM. This extension is defined as the mean reverting form of the state space model and is divided into two equations: the observation equation (Eq. 4) and the state equation (Eq. 5) (Rosenberg 1973). The unknown parameters of these equations are estimated via the Kalman filter algorithm (Kalman 1960), which is a widely used method for the linear state space model and is referred to here as the KFMR. It is expressed as follows.

4 $${R}_{it}-{R}_{ft}={\alpha }_{i}+{\beta }_{imt}\left({R}_{mt}-{R}_{ft}\right)+{\varepsilon }_{it}\;\;\;\;\;\; {\varepsilon }_{it}\sim N(0,{H}_{i})$$

5 $${\beta }_{imt}={\overline{\beta }}_{im}+{\phi }_{i}\left({\beta }_{im\;t-1}-{\overline{\beta }}_{im}\right)+{w}_{it} \;\;\;\;\;\;\;{w}_{it}\sim N\left(0,{Q}_{i}\right)$$

where ${\overline{\beta }}_{im}=\frac{1}{T}{\sum }_{t=1}^{T}{\beta }_{imt}$. Here, ${\varepsilon }_{it}$ and ${w}_{it}$ are assumed to be mutually independent residuals and normally distributed with mean 0 and variances ${H}_{i}$ and ${Q}_{i}$, respectively. ${\phi }_{i}$ evaluates the temporal autocorrelation in ${\beta }_{imt}$ in cryptocurrency *i* $\left(i=1,\dots ,10\right)$. Note that if ${\phi }_{i}=1$, this model becomes a random walk form of the state space model (Samuelson 1965), while if ${\phi }_{i}=0$, the model becomes a random coefficient form of the state space model (Schaefer et al. 1975). The prior parameter of KFMR is defined as follows.

6 $${\beta }_{im0 }\sim N\left({\mu }_{{\beta }_{im}},{\Sigma }_{{\beta }_{im}}\right)$$

Here, ${\mu }_{{\beta }_{im}}$ and ${\Sigma }_{{\beta }_{im}}$ are the initial estimates for ${\beta }_{im0}$ and are derived from the data in the estimation process. Optimal, updated, one-step-ahead, linear, and unbiased estimators of the unobservable state ${\beta }_{imt}$ are here provided by the Kalman filter algorithm. Briefly, the Kalman filter is a recursive algorithm that produces, at each time *t,* an estimator of the state vector ${\beta }_{imt}$, which is given by the orthogonal projection of the state vector onto the observed variables up to that time (Costa and Monteiro 2016).

The linear state space model via the Kalman filter and smoother algorithm (Shumway and Stoffer (2006) and Neslihanoglu et al. (2021)) is used to estimate the Tv-LMM in this study. This model is defined as follows:

7 $${Y}_{t}={A}_{t}{\kappa }_{t}+{\varepsilon }_{t}\;\;\;\;\;\;{\varepsilon }_{t} \sim N\left(0, H\right)$$

8 $${\kappa }_{t}=\Phi {\kappa }_{t-1}+{w}_{t}\;\;\;\;\; {w}_{t} \sim N\left(0, Q\right)$$

Let ${Y}_{t}$ be a $q \times 1$ vector of observations and ${\text{A}}_{t}$ be a $q \times p$ observation matrix and ${\kappa }_{t}$ is a $p \times 1$ unobserved state vector at each time $t \;(t=1,\dots ,n).$ Here, the transition parameter, $\Phi$, is a $p \times p$ matrix. Let ${\varepsilon }_{t}$ be a $q \times 1$ vector of observation residuals, which are independent and identically distributed with ${\varepsilon }_{t} \sim N\left(0, H\right)$, and ${w}_{t}$ is a $p \times 1$ vector of state residuals independent and identically distributed with ${w}_{t} \sim N\left(0, Q\right).$ Here, the model is wholly identified with the use of two other assumptions. First, it is assumed that ${\varepsilon }_{t}$ and ${w}_{t}$ are mutually independent for all *t.* Second, it is assumed that the initial state vector is ${\kappa }_{0}\sim N({\mu }_{0},{\Sigma }_{0})$.

The main purpose of this procedure is to estimate for the unobserved state vector, ${\kappa }_{t}$, at time $t$ given ${Y}_{t}=\left\{{Y}_{1},{Y}_{2},\cdots ,{Y}_{n}\right\}$ at time *n*. Throughout this procedure, a prediction problem occurs when $t > n$; a filtering problem occurs when $t = n$; and a smoothing problem occurs when $t < n$. To solve these problems, the Kalman filter and smoother algorithms are used. These are defined below.

The forward recursion steps of the Kalman filter and smoother algorithm with initial conditions ${\kappa }_{0}^{0}={\mu }_{0}$ and ${P}_{0}^{0}={\Sigma }_{0}$, for $t=1,\dots ,n$ can be implemented to mitigate the prediction ($t > n$) and filtering ($t= n$) problems. These steps are outlined as follows.

Prediction steps:

9 $$\text{Set the state prediction }\;\;\;\;\; {\kappa }_{t}^{t-1}={\Phi \kappa }_{t-1}^{t-1}$$

10 $$\text{Set the state variance prediction } \;\;\;\;\;{P}_{t}^{t-1}={\Phi P}_{t-1}^{t-1}{\Phi }^{^{\prime}}+Q$$

Filtering steps:

11 $$\text{Set the innovations }\;\;\;\;\;{v}_{t}={Y}_{t}-{A}_{t}{\kappa }_{t}^{t-1}$$

12 $${\text{Set the variance matrices of innovations }}\;\;\;\;\; \Sigma_{t}=Var\left({v}_{t}\right)={A}_{t}{P}_{t}^{t-1}{\text{A}}_{t}^{^{\prime}}+H$$

13 $$\text{Set the Kalman gain } \;\;\;\;\;{K}_{t}={P}_{t}^{t-1}{\text{A}}_{t}^{^{\prime}}{\Sigma }_{t}^{-1}$$

14 $$\text{Set the state filtering }\;\;\;\;\;{\kappa }_{t}^{t}={\kappa }_{t}^{t-1}+{K}_{t}{v}_{t}$$

15 $$\text{Set the state variance filtering }\;\;\;\;\;{P}_{t}^{t}={P}_{t}^{t-1}-{K}_{t}{A}_{t}{P}_{t}^{t-1}$$

Equations (9) to (15) should be cycled through for each time *t*. The forward recursions in Eqs. (11) through (15) identify the Kalman filter.

The backward recursion steps of the Kalman filter and smoother algorithm with initial conditions ${\kappa }_{n}^{n}$ (Eq. 14) and ${P}_{n}^{n}$ (Eq. 15), which are obtained from the Kalman filter with $t=n$, for $t=n,n-1,\dots ,1$, can be used in order to mitigate the smoothing $\left(t<n\right)$ problem. This is shown as follows.

Smoothing steps:

16 $$\text{Set the smoothed state }\;\;\;\;\;{\kappa }_{t-1}^{n}={\kappa }_{t-1}^{t-1}+{J}_{t-1}\left({\kappa }_{t}^{n}-{\kappa }_{t}^{t-1}\right)$$

17 $$\begin{aligned}&\text{Set the smoothed error variance}\quad{P}_{t-1}^{n}={P}_{t-1}^{t-1}+{J}_{t-1}({P}_{t}^{n}-{P}_{t}^{t-1}){J}_{t-1}^{\prime} \\ & {}\text{where}\quad{J}_{t-1}={P}_{t-1}^{t-1}{\Phi }^{{\prime}}{[{P}_{t}^{t-1}]}^{-1}\end{aligned}$$

Equations (16) to (17) should be cycled through for each time *t*. The backward recursion that occurs in Eqs. (16) through (17) is called the Kalman smoother. To estimate a state ${\kappa }_{t}$ given ${Y}_{n}$ with $t<n$, the Kalman filter is applied recursively until reaching state${\kappa }_{n}$. While continuing through the cycle, the values ${\kappa }_{t}^{t-1}$, ${\kappa }_{t}^{t}$, ${P}_{t}^{t-1}$, and ${P}_{t}^{t}$ ($t=1,\dots ,n$,) are stored. Next, one works backwards by applying the Kalman smoother until one reaches the state,$t$, which one is trying to estimate for.

Throughout this process, one makes the assumption that the system matrices ($H, \Phi$ and $Q$) and the initial mean ${\mu }_{0}$ and variance ${\Sigma }_{0}$, are both known. As occurs more often, however, some of the system matrices elements depend on an unknown parameters vector, Θ. One estimates the unknown parameters vector, Θ, by calculating for maximum likelihood. The loglikelihood function of the linear state space model (Eqs. (7) and (8)), which was coined by Harvey (1989) as “prediction error decomposition,” is stipulated as follows.

18 $$\text{log}{L}_{Y}\left(\Theta \right)= -\frac{nq}{2}\text{log}\left(2\pi \right)-\frac{1}{2}\sum_{t=1}^{n}\text{log}\left|{\Sigma }_{t}\left(\Theta \right)\right|-\frac{1}{2}\sum_{t=1}^{n}{{v}_{t}\left(\Theta \right)}^{^{\prime}}{\Sigma }_{t}{\left(\Theta \right)}^{-1}{v}_{t}\left(\Theta \right)$$

Here, $${v}_{t}\left(\Theta \right)$$ and $${\Sigma }_{t}\left(\Theta \right)$$ are calculated routinely by the Kalman filter (Eqs. 9–15) by assuming that $${\Sigma }_{t}\left(\Theta \right)$$ is nonsingular for *t* = *1,…,n*. The Newton–Raphson algorithm can be utilized successively for the purpose of updating the parameter values until the loglikelihood function (Eq. 18) is enlarged to its maximum level. The standard deviation of the unknown parameters vector, $$\Theta$$, is here calculated by $$\sqrt{diag\left(\Theta \left({H\left(\Theta \right)}^{-1}\right)\Theta \right)}$$ where $$H(\Theta )=\frac{{\partial }^{2}log{L}_{Y}\left(\Theta \right)}{\partial\Theta \partial {\Theta }^{^{\prime}}}$$ (known as Hessian matrix$$)$$, obtained in the Newton–Raphson algorithm. The *optim* package in the *R* software (R Core Team 2018) is used for the Newton–Raphson algorithm. Refer to Durbin and Koopman (2012) for an exhaustive review of the loglikelihood function.

The mechanism by which the Tv-LMM is applied, which is based on the Kalman filter and smoother algorithm, is related above. Throughout this procedure, the system matrices ($H, \Phi$ and $Q$) and the initial mean ${\mu }_{0}$ and variance ${\Sigma }_{0}$ of Tv-LMM model (Eqs. (3) and (4)) are adapted as follows.

19 $$\begin{aligned} & \Phi =\left[\begin{array}{cc}0& 0\\ 0& {\phi }_{i}\end{array}\right]\quad Q=\left[\begin{array}{cc}0& 0\\ 0& {Q}_{i}\end{array}\right] H={H}_{i}\\ & {\kappa }_{0}^{0}={\left[\begin{array}{cc}{\mu }_{{{\upalpha }}_{{\rm i}0}} & {\mu }_{{\upbeta }_{{\rm im}0}}\end{array}\right]}^{^{\prime}} \quad{P}_{0}^{0}=\left[\begin{array}{cc}{\Sigma }_{{\alpha }_{{\rm i}0}}& 0\\ 0& {\Sigma }_{{\upbeta }_{{\rm im}0}}\end{array}\right]\end{aligned}$$

In addition, the unknown parameters vector, $\Theta =\left\{{Q}_{i}{,{H}_{i}, \phi }_{i},{{\upalpha }}_{{\rm i}, },\stackrel{-}{{\beta }_{i}}\right\}$, is estimated by means of a consistent application of the Kalman Filter. The parameter estimation procedure and the *R* software coding (R Core Team 2018) of Kalman filter and smoother are briefly outlined in Shumway and Stoffer (2006).

The performance of the proposed models is compared by utilizing the MAE and the MSE criteria, represented as follows.

20 $$\text{MAE}=\frac{1}{T}\sum_{t=1}^{T}\left|\left(\widehat{{R}_{it}-{R}_{ft}}\right)-\left({R}_{it}-{R}_{ft}\right)\right|$$

21 $$\text{MSE}=\frac{1}{T}\sum_{t=1}^{T}{\left(\left(\widehat{{R}_{it}-{R}_{ft}}\right)-\left({R}_{it}-{R}_{ft}\right)\right)}^{2}$$

According to these criteria, the models with the lowest MSE and MAE values proffer a better modeling (forecasting) performance.

The Diebold–Mariano (DM) test (Diebold and Mariano 1995) is here used for the robustness checking of the model fit (forecasting) accuracy in the two aforementioned models in terms of the MAE and MSE as the measures of the in-sample model fitting (out-of-sample forecasting) procedure, represented as follows.

22 $$\text{DM}=\frac{\overline{d }}{Var\left(\overline{d }\right)}$$

$${d=\left|{\left(\left(\widehat{{R}_{it}-{R}_{ft}}\right)-\left({R}_{it}-{R}_{ft}\right)\right)}_{a}\right|}^{w}-{\left|{\left(\left(\widehat{{R}_{it}-{R}_{ft}}\right)-\left({R}_{it}-{R}_{ft}\right)\right)}_{b}\right|}^{w}$$

Here, ${\left(\left(\widehat{{R}_{it}-{R}_{ft}}\right)-\left({R}_{it}-{R}_{ft}\right)\right)}_{a}$ and ${\left(\left(\widehat{{R}_{it}-{R}_{ft}}\right)-\left({R}_{it}-{R}_{ft}\right)\right)}_{b}$ are the residuals for the two aforementioned models, that is, *a* and *b* for the *t*^th^ (*t* = 1*,…,T*). According to Choudhry and Wu (2009), *w is* defined as 1 for MAE and 2 for MSE. In the DM test, given the null hypothesis of no difference in levels of model fit (forecasting) accuracy between the two models, the DM test statistic follows a ${t}_{n-1}$ (Neslihanoglu et al. 2020). Note that these analyses were computed using the *R* software (R Core Team 2018).

## Results

### Model comparison

#### In-sample model fit

The comparison of the proposed models’ performance for modeling each cryptocurrency return in the pre-COVID-19 and COVID-19 periods uses the in-sample model fitting procedure. The MSE and MAE results between the actual and the theoretical values of the model for each cryptocurrency return during both time periods are summarized, respectively, in Table 3.

**Table 3:** In-sample model fit comparison criteria

Criteria	MAE × 10^2^					
Period	Pre-COVID-19			COVID-19		
Model	LMM	GAM	Tv-LMM	LMM	GAM	Tv-LMM
BPI	1.026	0.995	**0.693**	0.977	0.947	**0.498**
ETH	1.056	1.047	**0.710**	1.061	1.017	**0.594**
XRP	1.231	1.231	**0.765**	1.003	1.004	**0.776**
BCH	1.631	1.560	**1.162**	1.267	1.228	**1.008**
BNB	2.171	2.162	**1.996**	1.623	1.606	**1.599**
LINK	3.843	3.818	**3.694**	3.006	2.997	**2.387**
CRO	3.602	3.602	**3.600**	1.777	1.772	**1.654**
LTC	1.580	1.573	**1.321**	1.141	1.101	**0.910**
BSV	3.073	2.844	**2.145**	1.614	1.521	**0.783**
ADA	1.651	1.640	**1.517**	1.920	1.911	**1.810**
Average	2.086	2.047	**1.760**	1.539	1.510	**1.202**

Criteria	MSE × 10^4^					
Period	Pre-COVID-19			COVID-19		
Model	LMM	GAM	Tv-LMM	LMM	GAM	Tv-LMM
BPI	2.373	2.245	**1.058**	1.748	1.692	**0.476**
ETH	2.532	2.485	**1.134**	2.163	2.003	**0.694**
XRP	3.793	3.689	**1.330**	2.291	2.291	**1.414**
BCH	6.765	6.140	**3.410**	3.135	2.944	**1.979**
BNB	8.923	8.756	**7.625**	6.151	6.071	**6.030**
LINK	31.317	30.971	**29.183**	18.686	18.521	**11.819**
CRO	51.187	51.187	**51.180**	6.543	6.511	**5.641**
LTC	5.296	5.260	**3.445**	2.618	2.433	**1.597**
BSV	38.860	30.440	**18.874**	5.975	5.375	**1.322**
ADA	5.181	5.001	**4.300**	8.538	8.468	**7.484**
Average	15.623	14.617	**12.154**	5.785	5.631	**3.846**

Bold values indicate the best modeling performance for cryptocurrency price in terms of the lowest MAE and MSE, respectively

As shown in Table 3, on average, the TV-LMM (with the lowest MAE and MSE) improves on the LMM (with the highest MAE and MSE) in terms of MAE (MSE) by 15.6% (22.2%) for cryptocurrency in the pre-COVID-19 period, while it improves on LMM by 21.9% (33.5%) for cryptocurrency during the COVID-19 period. In addition, on average, the GAM improves on the LMM in terms of MAE (MSE) by 1.9% (6.4%) for cryptocurrency in the pre-COVID-19 period and by 1.9% (2.7%) for cryptocurrency during the COVID-19 period. Clearly, the performance of all models with relation to cryptocurrencies in the pre-COVID-19 period is worse than their performance for cryptocurrencies during the COVID-19 period. This may be because cryptocurrencies during the COVID-19 period are more stable than in the pre-COVID-19 period, as evidenced by Table 2. To sum up, the Tv-LMM model appears to be more desirable for modeling the daily cryptocurrency price indices in the pre-COVID-19 and COVID-19 periods, seeing as it achieves the lowest average MAE and MSE.

### Out-of-sample forecasting

An assessment of the aforementioned models’ forecasting performance is conducted here utilizing 1-day and 7-day ahead predictions with the rolling window technique for both the pre-COVID 19 and COVID-19 periods. To do this, the length of the rolling window in both periods is 180 days (6 months), and the length of the prediction period is 30 days (1 month) to predict ${\beta }_{it}$ by generating a 1-day ahead and 7-day ahead forecast, respectively, for each cryptocurrency during these periods. The MSE and MAE results between the actual and the predicted values of returns of each cryptocurrency over these 30 values during both time periods are summarized in Tables 4 and 5, respectively.

**Table 4:** Out-of-sample forecasting comparison criteria (1-day ahead forward prediction)

Criteria	MAE × 10^2^					
Period	Pre-COVID-19			COVID-19		
Model	LMM	GAM	Tv-LMM	LMM	GAM	Tv-LMM
BPI	0.758	0.834	**0.429**	0.981	0.956	**0.613**
ETH	1.560	1.584	**0.891**	0.684	0.657	**0.486**
XRP	1.472	1.409	**0.959**	0.859	0.855	**0.731**
BCH	1.232	1.450	**0.673**	1.360	1.361	**1.188**
BNB	1.502	1.415	**1.219**	1.459	1.470	**1.385**
LINK	3.995	3.816	**3.442**	1.802	1.796	**1.438**
CRO	1.838	1.802	**1.640**	2.899	2.899	**2.778**
LTC	1.133	1.189	**0.690**	1.404	1.398	**1.099**
BSV	3.423	2.740	**1.842**	1.106	1.103	**0.765**
ADA	1.327	1.388	**0.817**	1.201	1.201	**1.143**
Average	1.824	1.763	**1.260**	1.376	1.370	**1.163**

Criteria	MSE × 10^4^					
Period	Pre-COVID-19			COVID-19		
Model	LMM	GAM	Tv-LMM	LMM	GAM	Tv-LMM
BPI	0.983	1.072	**0.344**	1.745	1.627	**0.755**
ETH	4.787	4.626	**1.524**	0.730	0.718	**0.411**
XRP	4.490	4.175	**2.698**	1.413	1.410	**1.078**
BCH	2.522	3.221	**0.899**	4.074	3.867	**3.123**
BNB	3.971	3.761	**3.161**	3.994	3.965	**3.626**
LINK	25.729	23.759	**18.416**	5.934	5.935	**3.407**
CRO	6.436	6.068	**4.981**	19.099	19.099	**17.194**
LTC	2.851	2.828	**1.131**	3.862	3.816	**2.049**
BSV	19.631	14.336	**8.556**	2.423	2.421	**1.273**
ADA	2.794	2.830	**1.123**	2.679	2.679	**2.364**
Average	7.419	6.668	**4.283**	4.595	4.554	**3.528**

Bold values indicate the best predictability performance for cryptocurrency price in terms of the lowest MAE and MSE, respectively

**Table 5:** Out-of-sample forecasting comparison criteria (7-day ahead forward prediction)

Criteria	MAE × 10^2^					
Period	Pre-COVID-19			COVID-19		
Model	LMM	GAM	Tv-LMM	LMM	GAM	Tv-LMM
BPI	0.707	0.785	**0.404**	0.898	0.883	**0.526**
ETH	1.530	1.560	**0.921**	0.628	0.607	**0.423**
XRP	1.467	1.419	**1.022**	0.844	0.840	**0.714**
BCH	1.419	1.592	**0.699**	1.311	1.324	**1.143**
BNB	1.782	1.714	**1.547**	1.628	1.626	**1.533**
LINK	4.173	4.057	**3.708**	1.925	1.917	**1.544**
CRO	1.777	1.730	**1.592**	2.721	2.721	**2.576**
LTC	1.217	1.231	**0.744**	1.374	1.384	**1.067**
BSV	3.621	2.982	**2.161**	1.156	1.157	**0.777**
ADA	1.278	1.391	**0.843**	1.147	1.147	**1.092**
Average	1.897	1.846	**1.364**	1.363	1.361	**1.140**

Criteria	MSE × 10^4^					
Period	Pre-COVID-19			COVID-19		
Model	LMM	GAM	Tv-LMM	LMM	GAM	Tv-LMM
BPI	0.915	0.997	**0.326**	1.572	1.484	**0.599**
ETH	4.624	4.562	**1.655**	0.648	0.630	**0.311**
XRP	4.502	4.268	**2.969**	1.405	1.405	**1.061**
BCH	3.416	3.974	**0.978**	3.890	3.694	**3.004**
BNB	6.407	5.970	**4.974**	4.848	4.800	**4.376**
LINK	28.587	26.649	**21.910**	6.483	6.455	**3.806**
CRO	6.227	5.890	**4.811**	17.441	17.441	**15.062**
LTC	3.100	3.042	**1.224**	3.681	3.700	**1.869**
BSV	21.565	17.346	**11.769**	2.588	2.593	**1.323**
ADA	2.585	2.809	**1.064**	2.333	2.333	**2.067**
Average	8.193	7.551	**5.168**	4.489	4.454	**3.348**

Bold values indicate the best predictability performance for cryptocurrency price in terms of the lowest MAE and MSE, respectively

As shown in Tables 4 and 5, on average, the TV-LMM (with the lowest MAE and MSE) improves on the LMM (with the highest MAE and MSE) in terms of MAE (MSE) by 30.9% (42.3%) for the 1-day ahead forecast and by 28.1% (36.9%) for the 7-day ahead forecast for cryptocurrencies in the pre-COVID-19 period. It improves on the LMM by 15.5% (23.2%) for the 1-day ahead forecast and by 16.4% (25.4%) for the 7-day ahead forecast for cryptocurrencies during the COVID-19 period. Moreover, on average, the GAM improves on the LMM in terms of MAE (MSE) by 3.4% (10.1%) for the 1-day ahead forecast and by 2.7% (7.8%) for the 7-day ahead forecast for cryptocurrency in the pre-COVID-19 period. On average, it improves on the LMM by 0.4% (0.9%) for the 1-day ahead forecast and by 0.2% (0.8%) for the 7-day ahead forecast for cryptocurrencies during the COVID-19 period. It is also apparent that all three models’ cryptocurrency results in the 1-day ahead and 7-day ahead forecast procedures during the COVID-19 period are better than those in the 1-day ahead and 7-day ahead forecast procedures in the pre-COVID-19 period. This may be due to the fact that the cryptocurrencies were more subject to outliers in the pre-COVID-19 period than in the COVID-19 period. To summarize, Tv-LMM seems preferable when predicting the daily cryptocurrency price indices in the pre-COVID-19 and the COVID-19 periods given that it generated the lowest average MSE and MAE.

#### Robustness check

The robustness check of the model fitting and forecasting accuracy of the two aforementioned models using the DM test described in Sect. 3 in terms of MAE and MSE for both procedures and the 10 cryptocurrencies in the pre-COVID-19 and COVID-19 periods are discussed in this section, respectively. According to the DM test, the null hypothesis states that no differences exist in levels of model fitting (forecasting) accuracy between the two models in the in-sample (out-of-sample) procedure for each cryptocurrency in each time period. Tables 6 and 7 show the numbers of cryptocurrencies that reject the null hypothesis by using the DM test at the 5% significance level during the pre-COVID 19 and COVID-19 periods, respectively.

**Table 6:** The numbers of cryptocurrencies that reject the null hypothesis in terms of the MAE and MSE criteria in-sample procedure during the pre-COVID-19 and COVID-19 periods

Period	Pre-COVID-19		COVID-19	
Model	GAM	Tv-LMM	GAM	Tv-LMM
MAE				
LMM	6	8	8	8
GAM		9		9
MSE				
LMM	5	8	7	8
GAM		9		9

**Table 7:** The numbers of cryptocurrencies that reject the null hypothesis in terms of the MAE and MSE criteria out-of-sample procedure during the pre-COVID-19 and COVID-19 periods

Period	Pre-COVID-19			COVID-19	
Model	GAM	Tv-LMM		GAM	Tv-LMM
*1-day ahead forecast*					
MAE					
LMM	2	10		1	7
GAM		8			7
MSE					
LMM	2		6	1	7
GAM			6		7
*7-day ahead forecast*					
MAE					
LMM	8	10		3	10
GAM		10			10
MSE					
LMM	7		10	3	10
GAM			10		10

As shown in Tables 6 and 7, the Tv-LMM (which exhibits the best model fitting and forecasting performance) is statistically significant at the different levels of modelling for the 1-day and 7-day ahead forecast accuracy for the LMM and GAM in terms of MAE and MSE for both procedures during the pre-COVID-19 and COVID-19 periods. Moreover, GAM is not generally statistically significant at the different levels of forecasting accuracy for LMM in each time period. In sum, the Tv-LMM seems to be the preferable model for cryptocurrencies in the pre-COVID-19 and COVID-19 periods.

#### Graphical summary

**Fig. 3:**
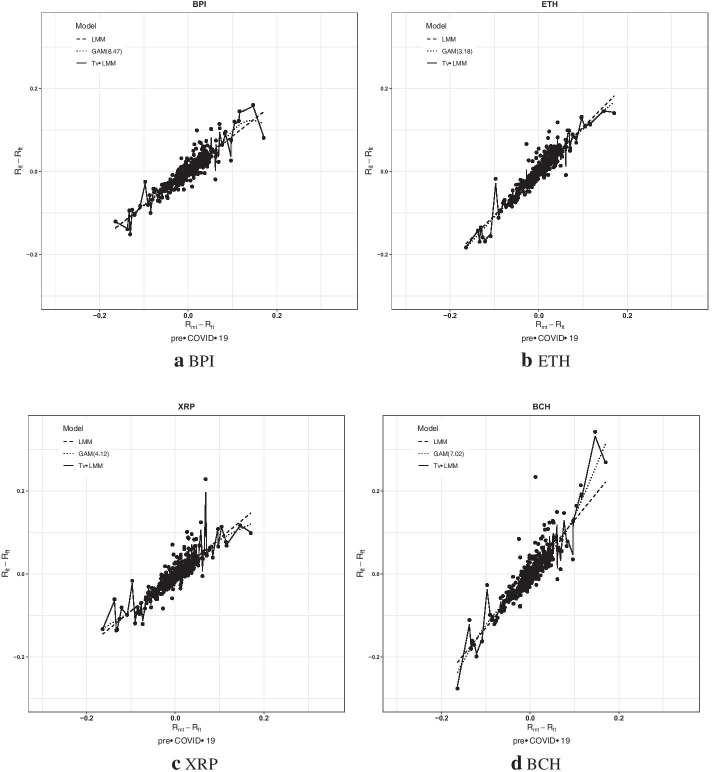
The scatter plots of the four cryptocurrencies’ daily excess returns in the pre-COVID-19 period

**Fig. 4:**
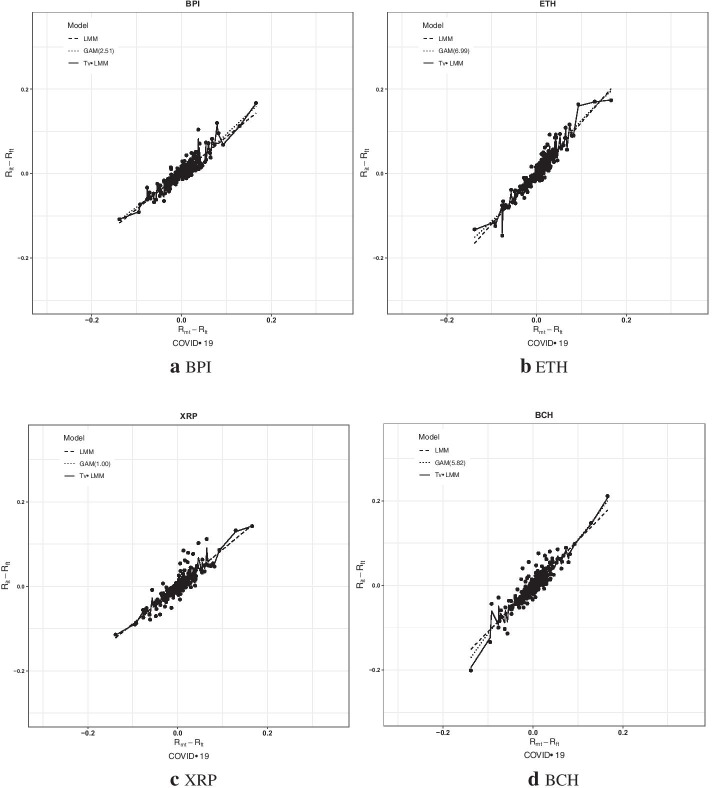
The scatter plots of the four cryptocurrencies’ daily excess returns in the COVID-19 period

The aforementioned models’ modeling performance is also presented using scatter plots to show the relationship between the returns of each cryptocurrency and the CCI30 during the pre-COVID-19 and COVID-19 periods. For the sake of brevity, the top four cryptocurrencies by adjusted market capitalization are BPI, ETH, XRP, and BCH; their fitted model plots for the pre-COVID-19 and COVID-19 periods are represented in Figs. 3 and 4, respectively.

As shown in Figs. 3 and 4, the Tv-LMM provides a much closer fit with the cryptocurrency data (especially for the COVID-19 period) owing to the time-varying relationship estimated between the excess returns of the cryptocurrency and the CCI30, suggesting that the short-term volatility in this relationship is captured by the Tv-LMM. The estimated relationships between the excess returns of the cryptocurrencies and the CCI30 from the LMM and the GAM are generally close to each other, with the exception of extreme values, which are more commonly observed in the pre-COVID-19 period than in the COVID-19 period. To sum up, the Tv-LMM seems to be the most appropriate model for cryptocurrencies, especially for the COVID-19 period.

#### Best model fit and forecasting performance

The model with the best modeling and forecasting performance for the pre-COVID 19 and COVID-19 periods is the TV-LMM via KFMR parameter estimates, which are described in Sect. 2 for each cryptocurrency and each time period. These are summarized in Tables 8 and 9, respectively.

**Table 8:** Tv-LMM via KFMR parameter estimates (with *standard errors*) of cryptocurrencies during the pre-COVID-19 period

Period	Pre-COVID-19					
Cryptocurrencies	$\widehat{{\varvec{Q}}_{\varvec{i}}}$	$\widehat{{\varvec{H}}_{\varvec{i}}}$	$\widehat{{\varvec{\phi }}_{\varvec{i}}}$	$\widehat{{\varvec{\alpha} }_{\varvec{i}}}$	$\widehat{\stackrel{-}{{\varvec{\beta} }_{\varvec{i}}}}$	Adjusted ${\varvec{R}}^{2}$
BPI	0.0586	0.0001	0.5214	0.0006	0.8314	0.910
	*(0.0260)*	*(0.0000)*	*(0.2233)*	*(0.0000)*	*(0.0288)*	
ETH	0.1032	0.0002	0.0000	0.0002	1.0663	0.936
	*(0.0300)*	*(0.0000)*	*(0.0000)*	*(0.0000)*	*(0.0327)*	
XRP	0.1740	0.0002	0.1365	− 0.0019	0.9077	0.904
	*(0.0342)*	*(0.0000)*	*(0.0289)*	*(0.0000)*	*(0.0356)*	
BCH	0.1392	0.0004)	0.4928	− 0.0002	1.2472	0.883
	*(0.0457)*	*(0.0000)*	*(0.1421)*	*(0.0000)*	*(0.0682)*	
BNB	0.0145	0.0008	0.8856	0.0024	0.8971	0.589
	*(0.0133)*	*(0.0001)*	*(1.0437)*	*(0.0000)*	*(0.0614)*	
LINK	0.0880	0.0030	0.0000	0.0055	0.8043	0.256
	*(0.0882)*	*(0.0002)*	*(0.0000)*	*(0.0000)*	*(0.0665)*	
CRO	0.0000	0.0051	0.9338	0.0014	0.5635	0.182
	*(0.0000)*	*(0.0003)*	*(10.5574)*	*(0.0000)*	*(0.0558)*	
LTC	0.0476	0.0004	0.7528	− 0.0002	1.1859	0.850
	*(0.0266)*	*(0.0000)*	*(0.4850)*	*(0.0000)*	*(0.0665)*	
BSV	0.7893	0.0023	0.2965	− 0.0006	1.2477	0.708
	*(0.6982)*	*(0.0003)*	*(0.3516)*	*(0.0000)*	*(0.1394)*	
ADA	0.0115	0.0005	0.8645	− 0.0009	1.1272	0.799
	*(0.0093)*	*(0.0000)*	*(0.8842)*	*(0.0000)*	*(0.0595)*	
*Average*	*0.1426*	*0.0014*	*0.4884*	*0.0006*	*0.9878*	*0.702*

**Table 9:** Tv-LMM via KFMR parameter estimates (with *standard errors*) of cryptocurrencies during the COVID-19 period

Period	COVID-19					
Cryptocurrencies	$\widehat{{\varvec{Q}}_{\varvec{i}}}$	$\widehat{{\varvec{H}}_{\varvec{i}}}$	$\widehat{{\varvec{\phi} }_{\varvec{i}}}$	$\widehat{{\varvec{\alpha} }_{\varvec{i}}}$	$\widehat{\stackrel{-}{{\varvec{\beta} }_{\varvec{i}}}}$	Adjusted ${\varvec{R}}^{\varvec{2}}$
BPI	0.1302	0.0001	0.0000	0.0008	0.7931	0.952
	*(0.0308)*	*(0.0000)*	*(0.0000)*	*(0.0000)*	*(0.0316)*	
ETH	0.1387	0.0001	0.1340	− 0.0001	1.2245	0.962
	*(0.0468)*	*(0.0000)*	*(0.0471)*	*(0.0000)*	*(0.0563)*	
XRP	0.0667	0.0002	0.0000	− 0.0011	0.8822	0.871
	*(0.0346)*	*(0.0000)*	*(0.0000)*	*(0.0000)*	*(0.0353)*	
BCH	0.0680	0.0002	0.1519	− 0.0015	1.0349	0.878
	*(0.0291)*	*(0.0000)*	*(0.0705)*	*(0.0000)*	*(0.0477)*	
BNB	0.0006	0.0006	0.9571	0.0010	0.9955	0.666
	*(0.0018)*	*(0.0001)*	*(3.7163)*	*(0.0000)*	*(0.0746)*	
LINK	0.4621	0.0014	0.2840	0.0019	1.5011	0.713
	*(0.3672)*	*(0.0002)*	*(0.2164)*	*(0.0000)*	*(0.1837)*	
CRO	0.0153	0.0006	0.8692	0.0013	0.7841	0.593
	*(0.0236)*	*(0.0001)*	*(1.5318)*	*(0.0000)*	*(0.0695)*	
LTC	0.0445	0.0002	0.6379	− 0.0015	1.0879	0.902
	*(0.0275)*	*(0.0000)*	*(0.4091)*	*(0.0000)*	*(0.0596)*	
BSV	0.4123	0.0002	0.0000	− 0.0032	1.0777	0.936
	*(0.0910)*	*(0.0000)*	*(0.0000)*	*(0.0000)*	*(0.0737)*	
ADA	0.0119	0.0008	0.9189	0.0014	1.3375	0.721
	*(0.0126)*	*(0.0001)*	*(1.3400)*	*(0.0000)*	*(0.1558)*	
*Average*	*0.1350*	*0.0004*	*0.3953*	*− 0.0001*	*1.0719*	*0.819*

**Fig. 5:**
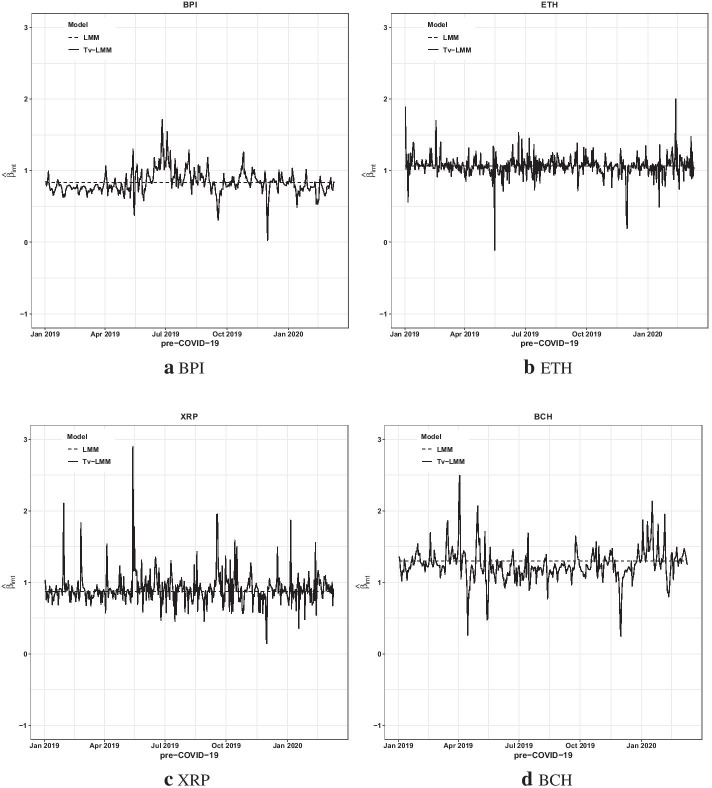
The estimated $\left({\widehat{\beta }}_{imt}\right)$ plots of four cryptocurrencies in the pre-COVID-19 period

**Fig. 6:**
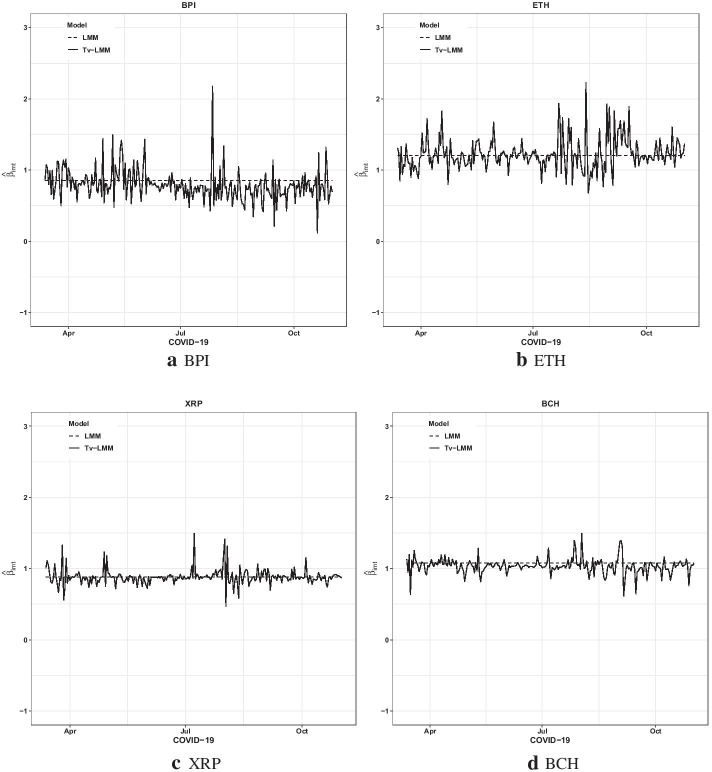
The estimated $\left({\widehat{\beta }}_{imt}\right)$ plots of four cryptocurrencies in the COVID-19 period

Tables 8 and 9 detail some key points. The average estimated variances of observation ($\widehat{{H}_{i}}$) and state ($\widehat{{Q}_{i}}$) values for the cryptocurrencies in the pre-COVID-19 period are higher than those during the COVID-19 period. This may be because of higher cryptocurrency volatilities in the pre-COVID-19 period compared to those in the COVID-19 period, as illustrated by Table 2. According to the average Adjusted *R*^*2*^, Tv-LMM provides a better performance during the COVID-19 period than in the pre-COVID-19 period. Moreover, the average temporal autocorrelation (captured by $\widehat{{\phi }_{i}}$) for the time-varying beta risk of cryptocurrencies is slightly higher in the pre-COVID-19 period than in the COVID-19 period. This proffers that the time-varying beta risk parameter changed rapidly in the pre-COVID-19 period, as illustrated by Figs. 5 and 6.

To provide additional information on the behavior of the beta risk estimates of the top four cryptocurrencies by adjusted market capitalization (BPI, ETH, XRP, and BCH), Figs. 5 and 6 present the cryptocurrencies’ beta risk estimates from the Tv-LMM (exhibiting the best performance) and LMM (exhibiting the worst performance) in both time periods, respectively. The time-varying beta risk $\left({\widehat{\beta }}_{imt}\right)$ estimates of the cryptocurrencies from the Tv-LMM fluctuate around those from the LMM, as expected, during both the pre-COVID-19 and COVID-19 periods.

Tables 8, 9 and 10 provide a guideline for active (alpha) and passive (beta) investors upon which their portfolios can be based and with which they can learn what risks and rewards each cryptocurrency may entail. The average alpha ($\widehat{{\alpha }_{i}}$) is close to 0 and positive in cryptocurrencies in the pre-COVID-19 period, while it is close to 0 and negative in cryptocurrencies during the COVID-19 period. This implies that the cryptocurrency *i* actual returns is higher than that of the expected returns derived by Tv-LMM in the pre-COVID-19 period, while it is lower than that of the expected returns derived by Tv-LMM during the COVID-19 period. It is worth mentioning that active (alpha) investors prefer to invest in cryptocurrencies with a positive alpha (being considered as valuable investments). Thus, it was observed that they preferred to make investments during the pre-COVID-19 period.

**Table 10:** Descriptive statistics of the time-varying beta risk $\left({\widehat{\beta }}_{imt}\right)$ estimates of the cryptocurrencies from the Tv-LMM during the pre-COVID-19 and COVID-19 periods

Period	Pre-COVID-19						
Cryptocurrencies	Minimum	1^st^ Quartile	Median	Mean	3^rd^ Quartile	Maximum	Standard deviation
BPI	0.0224	0.7491	0.8051	0.8314	0.8996	1.7152	0.1630
ETH	− 0.1134	1.0113	1.0657	1.0663	1.1153	2.0014	0.1622
XRP	0.1462	0.8052	0.8878	0.9077	0.9705	2.8997	0.2314
BCH	0.2440	1.1471	1.2268	1.2472	1.3335	2.4944	0.2264
BNB	0.4898	0.8085	0.9071	0.8971	1.0044	1.2676	0.1510
LINK	0.5391	0.7932	0.8043	0.8043	0.8168	1.1709	0.0529
CRO	0.5373	0.5635	0.5635	0.5635	0.5635	0.5635	0.0034
LTC	0.4932	1.0971	1.1773	1.1859	1.2683	2.3745	0.1996
BSV	− 0.9146	1.1074	1.2080	1.2477	1.3063	2.5686	0.2985
ADA	0.6222	1.0598	1.1413	1.1272	1.2046	1.4338	0.1234
*Average*	*0.2066*	*0.9142*	*0.9787*	*0.9878*	*1.0483*	*1.8490*	*0.1612*

Period	COVID-19						
Cryptocurrencies	Minimum	1^st^ Quartile	Median	Mean	3^rd^ Quartile	Maximum	Standard Deviation
BPI	0.1164	0.6779	0.7817	0.7931	0.8646	2.1763	0.2212
ETH	0.6799	1.1016	1.2048	1.2245	1.311	2.2274	0.2213
XRP	0.4677	0.8433	0.8781	0.8822	0.9012	1.4921	0.1106
BCH	0.6137	0.9998	1.0375	1.0349	1.0769	1.4932	0.1064
BNB	0.9473	0.9694	0.9869	0.9955	1.0295	1.0625	0.0344
LINK	0.4741	1.3404	1.4873	1.5011	1.6011	2.9460	0.3122
CRO	0.3541	0.7137	0.7741	0.7841	0.8823	1.0884	0.1393
LTC	0.8012	1.0123	1.0594	1.0879	1.1131	1.7902	0.1532
BSV	− 0.0645	0.9048	1.0548	1.0777	1.1867	2.0829	0.3395
ADA	1.0189	1.1904	1.3477	1.3375	1.4452	1.7919	0.1716
*Average*	*0.5409*	*0.9754*	*1.0612*	*1.0719*	*1.1412*	*1.8151*	*0.1810*

The average of the time-varying beta (${\widehat{\beta }}_{imt}$) (being a statistical measure of a cryptocurrency’s relative volatility to that of the CCI30, where it can be interpreted as both a measure of systematic risk and a performance measure) for cryptocurrencies is positive (1.0719); it was theoretically 7.19% more volatile than the CCI30 during the pre-COVID-19 period, and (0.9878), which is theoretically 1.22% less volatile than the CCI30 during the COVID-19 period. In addition, the fluctuation in time-varying betas is an indicator of the level of exposure to systematic risk. Thus, higher betas imply higher risk, while lower betas imply lower risk. Thus, cryptos with higher betas may gain more in up-markets but may also lose more in down-markets. BSV is the riskiest cryptocurrency with the highest range of time-varying beta risk ${\widehat{\beta }}_{imt}$ estimate series during both time periods. In the wake of the discussion about the stochastic behavior of cryptos’ betas, it can be concluded that passive investors’ (beta investor) investing preferences while allocating cryptos in their portfolios in the future may become more flexible and related to their risk-tolerance level.

## Conclusion

This research investigated the performance comparison of LMM and its extensions (namely, the GAM and the Tv-LMM), the model fitting, and the 1-day and 7-day ahead predictions for the 10 cryptocurrencies’ daily prices in the pre-COVID-19 and COVID-19 periods. The empirical findings in this research favor the Tv-LMM model, which outperforms others in both modeling and predicting the daily prices of these 10 cryptocurrencies, especially during the COVID-19 period. This provides evidence of a local linear relationship between each cryptocurrency with the CCI30 index as opposed to the traditional LMM, which provides evidence of global linearity.

The comparative analysis in this research clarifies the linearity extensions of the traditional market model in explaining cryptocurrency price. It is apparent that the time-varying linearity extension of the traditional market model, allowing for the time-varying beta risk parameters, absorbed the structural changes of the cryptocurrency price series. In conclusion, this research emphasizes the importance of the time-varying market model when dealing with crypto market inefficiencies.

These research findings should motivate a more systematic investigation of potential risk premia in the cryptocurrencies market as far as there exist specific types of cryptos with a higher exposure to systematic risk (beta risk). Thus, it would be interesting to investigate whether this is accounted for in the profitability of investors. The approach taken in this research suggests that a connection between the exposure to systematic risk and the gains accumulated by trading cryptos is likely to exist. This is strong evidence for the existence of crypto risk premiums. More analysis, however, is expected in this area. Nevertheless, the time dynamics of risk pricing casts doubt on the persistence of risk premiums during specific sub-periods, which indicates that there is more work to be done by regulators to improve the efficiency of this newly established market. More efforts should be put into further research to potentially improve the efficiency of this market, thereby allowing for the attraction of more investors and the risk aversion of their diverse portfolios by considering cryptos as well.

## Data Availability

The selected database to be considered by the model was found in the following data sources: Cryptocurrencies Index 30 (https://cci30.com), Bitcoin, Ethereum, XRP, Bitcoin Cash, Binance Coin, Chainlink, Crypto.com Coin, Litecoin, Bitcoin SV, Cardano Price Index (https://coinmarketcap.com), 1 month LIBOR rate (https://www.macrotrends.net).

## Abbreviations

LMM: Linear Market Model
OLS: Ordinary Least Squares
GAM: Generalized Additive Model
Tv-LMM: Time-varying Linear Market Model
KFMR: Mean-reverting form of State Space Model via Kalman Filter
MAE: Mean Absolute Error
MSE: Mean Squared Error
CCI30: Crypto Currency Index 30
BPI: Bitcoin Price Index
ETH: Ethereum Price Index
XRP: XRP Price Index
BCH: Bitcoin Cash Price Index
BNB: Binance Coin Price Index
LINK: Chainlink Price Index
CRO: Crypto.com Coin Price Index
LTC: Litecoin Price Index
BSV: Bitcoin SV Price Index
ADA: Cardano Price Index
R_f_: Risk-free rate
